# Efficacy and Safety of FX201, a Novel Intra-Articular IL-1Ra Gene Therapy for Osteoarthritis Treatment, in a Rat Model

**DOI:** 10.1089/hum.2021.131

**Published:** 2022-05-16

**Authors:** Rebecca Senter, Rogely Boyce, Marko Repic, Emily Walsh Martin, Monika Chabicovsky, Geneviève Langevin-Carpentier, Agathe Bédard, Neil Bodick

**Affiliations:** ^1^ Flexion Therapeutics, Inc.,^†^ Burlington, Massachusetts, USA; ^2^ Beechy Ridge ToxPath, LLC, Clay, West Virginia, USA; ^3^ MC Toxicology Consulting, Vienna, Austria; ^4^ Tremont Therapeutics, LLC, Charlestown, Massachusetts, USA; ^5^ Charles River Laboratories, Senneville, Canada; and; ^6^ Gate Science, Inc., Moultonborough, New Hampshire, USA.

**Keywords:** disease modification, FX201, gene therapy, immunogenicity, osteoarthritis, disease models > muscle/connective tissue/bone

## Abstract

Osteoarthritis (OA) is a disabling, degenerative disease characterized by progressive cartilage and bone damage. There remains a need for local therapies that, following a single injection, can provide long-term pain relief and functional improvement and potentially delay disease progression. FX201 is a novel, intra-articular (IA), interleukin-1 receptor antagonist (IL-1Ra) gene therapy in development for the treatment of OA. In this study, we assessed the efficacy, biodistribution, and safety of helper-dependent adenovirus (HDAd)-ratIL-1Ra, the rat surrogate of FX201, and the biodistribution of FX201, in the anterior cruciate ligament transection (ACLT) rat OA model. A single IA injection of HDAd-ratIL-1Ra administered 7 days post-ACLT mitigated OA-related changes to cartilage, bone, and the synovial membrane at week 12 following surgery. Furthermore, FX201 and HDAd-ratIL-1Ra persisted for at least 92 days in the injected joint and proximal tissues with minimal evidence of vector spreading peripherally. Finally, HDAd-ratIL-1Ra showed a favorable safety profile without any local or systemic adverse effects. In conclusion, HDAd-ratIL-1Ra demonstrated local therapeutic and disease-modifying effects and was well tolerated, supporting further clinical development of FX201.

## INTRODUCTION

Osteoarthritis (OA) is a painful and debilitating musculoskeletal disease that is characterized by intra-articular (IA) inflammation, deterioration of articular cartilage, and degenerative changes to periarticular and subchondral bone.^1,2^ Arthritis is the most common cause of disability in the United States and OA is the most common joint disease, affecting more than 30 million Americans, with numbers expected to grow as a result of aging, obesity, and sports injuries.^3–6^ OA commonly affects large weight-bearing joints like the knees and hips, but also occurs in the shoulders, hands, feet, and spine.^3^ Patients with OA suffer from joint pain, tenderness, stiffness, and limited movement.^4,5^ As the disease progresses, it becomes increasingly painful and debilitating, culminating in many cases in the need for total joint arthroplasty.^6^

Inflammation is believed to be a driving force behind the cartilage matrix breakdown, bone shape changes, and pain that occurs in OA.^5,6^ Among the proinflammatory cytokines, interleukin-1 (IL-1) is the most abundantly produced and contributes to these signs and symptoms.^7,8^ As such, controlling IL-1 signaling may be an advantageous therapeutic strategy that has the potential to produce both pain relief and disease-modifying activity.

The IL-1 family consists of two agonists, IL-1α and IL-1β; two receptors, biologically active IL-1 receptor type I (IL-1RI) and decoy IL-1 receptor type II (IL-1RII); and a receptor antagonist, interleukin 1 receptor antagonist (IL-1Ra). IL-1Ra is a true antagonist of the IL-1RI, competitively blocking the binding of both IL-1α and IL-1β, while having no inherent signaling capabilities itself. Local delivery of IL-1Ra to the joint decreases pain (as measured by lameness in horses),^9,10^ reduces cartilage damage,^8–19^ and reduces osteophyte size and prevalence.^8,9,14,18^ Moreover, the efficacy of IL-1Ra has been observed in mice, rats, rabbits, dogs, and horses, with delivery routes including direct injection of recombinant protein, transfer of plasmid DNA, and delivery of viral vectors containing the *IL-1Ra* gene.^8–19^

The clinical efficacy of IL-1Ra has been evaluated in knee and erosive hand OA, as well as acute ACL tear,^20–23^ where acute reductions in pain and functional improvements have been observed. This efficacy waned rapidly, likely due to the short half-life of IL-1Ra,^21^ indicating that a mechanism providing durable expression of IL-1Ra could be efficacious.

Gene therapy is a novel approach with the potential to provide long-term therapeutic protein expression, which could translate into durable pain relief and functional improvement and the potential to modify the disease. Previous pre-clinical studies in rats and dogs have shown the potential of gene therapy targeting cytokines to positively impact the OA disease process with a favorable safety profile.^24–26^ FX201 uses a helper-dependent adenovirus (HDAd) vector based on human serotype 5 (Ad5), which is designed to transfer a gene to cells in the joint to produce IL-1Ra under the control of an inflammation-sensitive promoter (unpublished data on file: Work was performed at Baylor College of Medicine, Houston, TX, in January 2013. Title of work was “*in vitro* expression of IL-1Ra from HDAd-eqIL-1Ra”). FX201 is in clinical development for the treatment of OA.

In this study, we evaluate the efficacy, biodistribution, and safety of HDAd-ratIL-1Ra, the rat surrogate of FX201, and the biodistribution of FX201, when administered as a single IA injection in the anterior cruciate ligament transection (ACLT) rat model of post-traumatic OA to establish a therapeutic index.

## MATERIALS AND METHODS

### Ethics

All procedures were conducted at Charles River Laboratories and approved by the Institutional Animal Care and Use Committee of Charles River Laboratories. Biodistribution and safety studies were conducted in accordance with Good Laboratory Practice guidelines.

### Animals and ACLT surgery

Male and female Sprague-Dawley rats (Charles River Canada, Inc.; Constant, Canada), 8–9 weeks of age at the initiation of all studies, were assigned to dose groups using a randomized stratification scheme designed to achieve comparable group mean body weights before ACLT surgery. All animals underwent surgery; animals in the sham arm underwent surgery, but did not undergo ACLT, and the remainder had ACLT surgery (Supplementary Fig. S1). In ACLT-operated rats, a parapatellar skin incision was made on the medial side of the right knee joint and the patellar tendon under isoflurane anesthesia.

The patella was then dislocated laterally to allow access into the joint space, and the ACL was transected in the flexed knee. The joint was irrigated with sterile saline to prevent ancillary inflammation, and a purpose-made suture was inserted. Buprenorphine was administered as an analgesic at least 30 min before surgery and 8 to 12 h after the first injection, and an injection of trimethoprim was provided at least 30 min before surgery and every 12 h.

### IA dosing of HDAd-ratIL-1Ra

IA dosing was based on synovial volume scaling between rat and human knee joints. Animals received IA injections of HDAd-ratIL-1Ra or vehicle (10 mM Tris buffer, 75 mM sodium chloride, 5% [w/v] sucrose, 0.02% [w/v] polysorbate-80, 1 mM magnesium chloride, 100 μM ethylenediaminetetraacetic acid, 0.5% [v/v] ethanol, and 10 mM His buffer [pH 7.4]) in the right knee joint under isoflurane anesthesia at a dose volume of 15 μL. Animals in the sham group did not receive an IA injection.

### Efficacy

To assess efficacy, rats were assigned to one of four study groups (sham: *n* = 10 and all other groups: *n* = 12) and received a single IA injection of HDAd-ratIL-1Ra (3.6 × 10^7^ or 3.1 × 10^8^ genome copies [GC]/dose) or vehicle 7 days following ACLT surgery (Supplementary Fig. S1A). Eleven weeks after dosing, rats were sacrificed, and whole right knee joints were harvested. Joints were decalcified in formic acid, embedded in paraffin, and sectioned coronally. For histopathological evaluation, cartilage and bone sections were stained with Safranin-O/Fast Green (SOFG), and synovial membrane sections were stained with hematoxylin and eosin.

The Osteoarthritis Research Society International (OARSI) grading system was used to score OA-associated structural changes, SOFG staining loss, clone formation, and chondrocyte loss in cartilage, bone, and the synovial membrane (Supplementary Table S1).^27,28^ For cartilage and bone, OARSI scores were generated for each of the four joint compartments: medial femur, lateral femur, medial tibia, and lateral tibia. Individual scores were recorded from representative sections, and composite scores (Supplementary Table S3) were generated from the sum of all individual scores (Supplementary Table S2).

### Biodistribution of FX201 and HDAd-ratIL-1Ra

To assess biodistribution as part of the Good Laboratory Practice (GLP) studies, male and female rats were equally assigned to one of three study groups (each *n* = 36) and were left untreated or given a single IA injection of FX201 or HDAd-ratIL-1Ra 28 days following surgery (Supplementary Fig. S1B). Rats from each group were sacrificed on days 8, 29, and 92 after dose (*n* = 12 [1:1 sex ratio per time point]), and biodistribution of FX201 and HDAd-ratIL-1Ra was assessed in tissues, including the brain, bone marrow, kidney, ovary, plasma, skin (at the injection site), and testis, using a validated quantitative polymerase chain reaction (qPCR) method.

In a companion study designed to further assess biodistribution in the whole knee joint versus key peripheral tissues, male rats were (*n* = 5 per dose level and time point) given a single IA injection of FX201. Animals were sacrificed at either 8 h or 8 days after dosing. Biodistribution was assessed using the same validated qPCR method in the injected whole knee joint (left knee joint), liver, iliac lymph node, popliteal lymph node, and right (un-injected) knee joint. Genomic DNA was purified from rat tissues using the QIAquick^®^ PCR Purification Kit (Qiagen, Hilden, Germany).

The qPCR reaction was performed using forward and reverse primers specific for the plasmid DNA (5′-AGACAGGAGGTCAGCATCTTTATTG-3′ and 5′-GACACAGACTCCGCTGTTATCAG-3′) and the TaqMan^®^-based qPCR assay (Applied Biosystems, Foster City, CA). A probe labeled with the reporter dye 6-carboxyfluorescein at the 5′ end and minor groove binding nonfluorescent quencher at the 3′ end (5′-CCCATGACACCTCTACAC-3′; Applied Biosystems) was used to detect the DNA amplicon. Standard qPCR thermocycling conditions were used, and qPCR data were collected using QuantStudio™ 7 Flex Real-Time PCR System/QuantStudio Real-Time PCR software version 1.4 (ThermoFisher Scientific, Waltham, MA).

### Safety

To assess safety, rats were assigned to one of six study groups (each *n* = 24) and received a single IA injection of HDAd-ratIL-1Ra or vehicle following ACLT surgery (Supplementary Fig. S1C). Mortality, clinical signs, in-life parameters, including body weight and food consumption, and clinical pathology were evaluated.

Serum samples were taken on days 29 and 92 after dosing and anti-Ad5 antibodies were quantified using a validated semiquantitative enzyme-linked immunosorbent assay. Anti-Ad5 antibodies were detected in rat serum samples with goat anti-rat immunoglobulin G and immunoglobulin M secondary antibodies conjugated to horseradish peroxidase (Jackson ImmunoResearch Laboratories, West Grove, PA). Rabbit anti-Ad5 antibody (Abcam, Cambridge, United Kingdom) and pooled rat serum (BioIVT; Westbury, NY) were used as positive and negative controls, respectively.

For analysis of T cell-mediated interferon-γ (IFN-γ) responses to Ad5 vector, spleens were collected from rats in all six study groups following euthanasia and processed to single-cell suspensions for enzyme-linked immunosorbent spot (ELISpot) analysis. A validated ELISpot method was used in which splenocytes were plated in triplicate at a density of 4.0 × 10^6^ viable cells/mL and stimulated with 3.0 nmol/mL AdV5 hexon peptide (Miltenyi Biotec, Bergisch Gladbach, Germany). Medium and phorbol myristate acetate/ionomycin (Millipore Sigma, St. Louis, MO) were included as negative and positive controls, respectively. IFN-γ was detected post-Ad5 peptide stimulation using a polyclonal biotinylated anti-rat IFN-γ antibody (R&D Systems; Minneapolis, MN).

Streptavidin-alkaline phosphatase and 5-bromo-4-chloro-3′ indolyl phosphate p-toluidine salt mixed with Nitro Blue tetrazolium chloride were then added to produce a chromogenic product that was detected in cells through an automated NucleoCounter. The number of spot-forming cells and the number of responding animals were counted for each study group.

### Statistical analysis

Descriptive statistics (*e.g.*, mean, median, and 95% confidence interval [CI]) were calculated for each study group. Differences between study groups were analyzed in Prism 8 (GraphPad, San Diego, CA) using either one- or two-way analysis of variance followed by Tukey's multiple comparisons test. For all statistical tests, *p* < 0.05 was considered significant.

## RESULTS

### HDAd-ratIL-1Ra decreased OA-induced joint damage in ACLT-operated rats

Disease-modifying activity of HDAd-ratIL-1Ra was assessed in rats given a single IA injection 7 days post-ACLT surgery. Microscopic changes in knee joints of untreated, sham-operated rats (*n* = 10) were limited to low incidence of superficial articular cartilage changes graded minimal in severity as assessed by surface irregularities with focal fibrillation/clefts/fissure, chondrocyte loss, and/or loss of SOFG staining.

All ACLT-operated rats (*n* = 36) developed characteristic minimal-to-severe OA microscopic changes in one or more of the examined articular compartments (medial femur, lateral femur, medial tibia, and lateral tibia) 12 weeks following ACLT surgery. These changes included surface irregularities to complete fibrillation/clefts/fissure/loss of articular cartilage, SOFG staining loss, clone formation (proliferation of chondrocytes), and/or chondrocyte loss.

**Figure 1.**
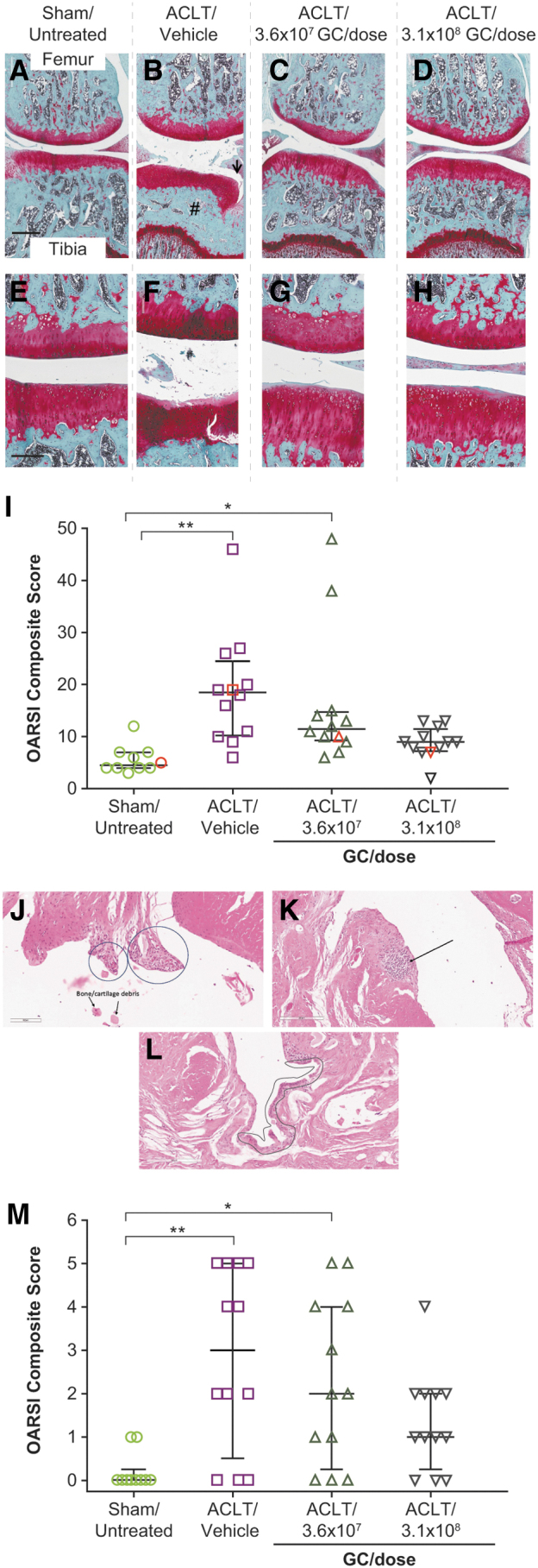
HDAd-ratIL-1Ra decreases OA-related joint damage in ACLT-operated rats. Eight- to nine-week-old male rats underwent ACLT surgery and received a single IA injection of HDAd-ratIL-1Ra or vehicle 7 days following surgery. Eleven weeks post-dosing, joints (*n* = 12 joints per group) were collected and stained with SOFG to assess OA-related changes to joint tissue. **(A–H)** Representative images of the lateral femur and tibia from each study group are shown at 4 × **(A–D)** and 10 × **(E–H)** magnification. **(B)** The *arrow* indicates fibrillation and fissuring of tibial cartilage, and the number sign (#) indicates subchondral bone remodeling. Scale bar represents 600 μm (4 × ) and 300 μm (10 × ). ACLT-induced fibrillation and fissuring of tibial cartilage and subchondral bone remodeling were substantially reduced with HDAd-ratIL-1Ra treatment compared with vehicle. Sham/untreated rats (*n* = 10 joints) were included as a control. **(I)** OARSI composite scores for cartilage and bone for each study group analyzed in **(A–D)** are shown. Individual animal data are shown; *whiskers* represent the interquartile range and data points in *red* are the joints shown in **(A–D)**. HDAd-ratIL-1Ra demonstrated dose-dependent decreases in composite scores compared with vehicle. **(J)** Representative image demonstrating villous hyperplasia with proliferation of fibroblasts/blood vessels and bone detritus (scale bar = 100 μm). **(K)** Representative image demonstrating lymphoplasmacytic infiltrates rarely forming aggregates or follicles (scale bar = 200 μm). **(L)** Representative image demonstrating synoviocyte proliferation/hypertrophy (scale bar = 200 μm). **(M)** OARSI composite (total) scores for hematoxylin and eosin-stained sections of the synovial membrane from each study group. HDAd-ratIL-1Ra demonstrated dose-dependent decreases in composite scores for OA-related changes to the synovial membrane compared with vehicle. *p < 0.05; **p < 0.01, one-way analysis of variance with Tukey's multiple comparisons test. Comparisons that are not denoted by * or ** were found to be nonsignificant (*p* ≥ 0.05). ACLT, anterior cruciate ligament transection; HDAd, helper-dependent adenovirus; IA, intra-articular; IL-1Ra, interleukin-1 receptor antagonist; OA, osteoarthritis; OARSI, Osteoarthritis Research Society International; SOFG, Safranin-O/Fast Green.

ACLT-induced fibrillation and fissuring of tibial cartilage and subchondral bone remodeling were substantially reduced in HDAd-ratIL-1Ra–treated rats compared with vehicle-treated rats (Fig. 1A–I) at 12 weeks after surgery, and improvement in ACLT-induced changes was observed with both HDAd-ratIL-1Ra doses (3.6 × 10^7^ and 3.1 × 10^8^ GC/dose). Decreases in OARSI composite scores for cartilage and bone were dose dependent.

HDAd-ratIL-1Ra also demonstrated dose-dependent decreases in composite scores of OA microscopic changes to the synovial membrane compared with vehicle, although differences were not statistically significant (Fig. 1J–M). All synovial microscopic findings were minimal in severity and included synoviocyte proliferation/hypertrophy, lymphoplasmacytic infiltrates rarely forming aggregates or follicles, villous hyperplasia with proliferation of fibroblasts/blood vessels, and cartilage and bone detritus.

**Figure 2.**
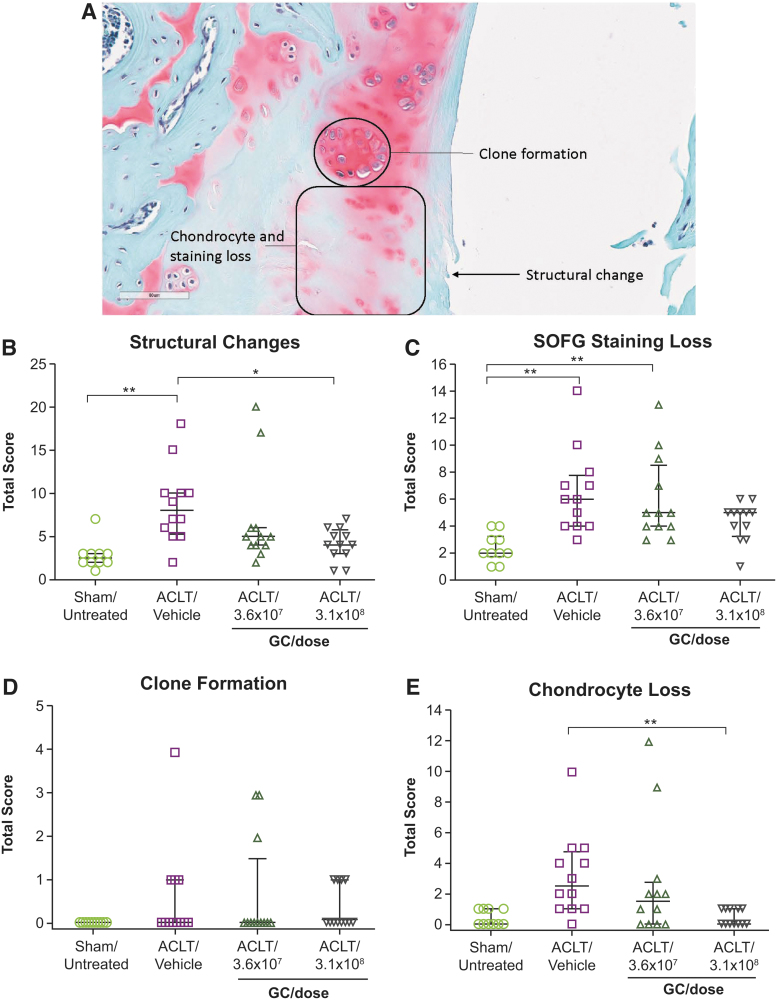
HDAd-ratIL-1Ra improves histopathological features of OA in ACLT-operated rats. ACLT surgery was performed on 8- to 9-week-old male rats to induce OA, and rats received a single IA injection of HDAd-ratIL-1Ra or vehicle 1 week following surgery. Eleven weeks later, joints were collected, sectioned, and stained with SOFG (*n* = 12 joints per group). Joints were evaluated for OA-like changes, including structural changes, SOFG staining loss, clone formation, and chondrocyte loss, as depicted in the representative vehicle-treated animal joint image **(A)**. Individual animal OARSI scores and median values for structural changes **(B)**, SOFG staining loss **(C)**, clone formation **(D)**, and chondrocyte loss **(E)** are shown; *whiskers* represent the interquartile range. Representative image from ACLT/Vehicle animal, scale bar represents 80 μm. Sham/untreated rats (*n* = 10 joints) were included as a control. Significant decreases in structural change and chondrocyte loss were observed with HDAd-ratIL-1Ra treatment (3.1 × 10^8^ GC/dose) compared with vehicle. However, decreases in severity of SOFG staining loss and clone formation with HDAd-ratIL-1Ra treatment compared with vehicle were not significant. *p < 0.05; **p < 0.01, one-way analysis of variance with Tukey's multiple comparisons test. Comparisons that are not denoted by * or ** were found to be nonsignificant (*p* ≥ 0.05). Average OARSI scores are presented in Supplementary Table S1.

Structural changes and chondrocyte loss were significantly reduced in ACLT-operated rats receiving HDAd-ratIL-1Ra at 3.1 × 10^8^ GC/dose compared with those receiving vehicle (*p* < 0.05, 95% CI, 0.14–8.53, and *p* < 0.05, 95% CI, 0.09–5.41, respectively) (Fig. 2A, B, E). Furthermore, slight decreases in severity of SOFG staining loss and incidence of clone formation were observed with HDAd-ratIL-1Ra treatment compared with vehicle at 12 weeks after surgery (Fig. 2A, C, D). Taken together, these results suggest that HDAd-ratIL-1Ra treatment decreased OA severity in ACLT-operated rats.

### FX201 predominantly remains in the knee joint following IA administration

Biodistribution of FX201 and HDAd-ratIL-1Ra vectors was initially assessed as part of the GLP toxicology evaluation on days 8, 29, and 92 after dosing in rats that had undergone ACLT 4 weeks before treatment (*n* = 12 per time point). The distribution profile of FX201 and HDAd-ratIL-1Ra was nearly identical and, as such, only data for FX201 are shown (Supplementary Fig. S3). As expected, FX201 vector concentrations were highest at the injection site (skin, synovial lavage, quadriceps femoris muscle, and draining iliac and popliteal lymph nodes), and vector DNA persisted for the duration of the 92-day study. Peripherally, FX201 was sporadically detected at low copy numbers in bone marrow, liver, lung, and spleen. Vector DNA was not detected in brain, heart, kidney, ovary, plasma, or testis.

Since treated joints in the GLP toxicity and biodistribution studies were used for histopathological assessment, evaluation of vector concentrations in the whole knee joint was not possible and a synovial fluid lavage was used instead to evaluate local persistence. As it would be expected that vector DNA would be present in local joint tissues rather than synovial fluid, we performed an additional companion study to evaluate the total vector genome concentrations in the whole knee joint following IA injection of FX201.

**Figure 3.**
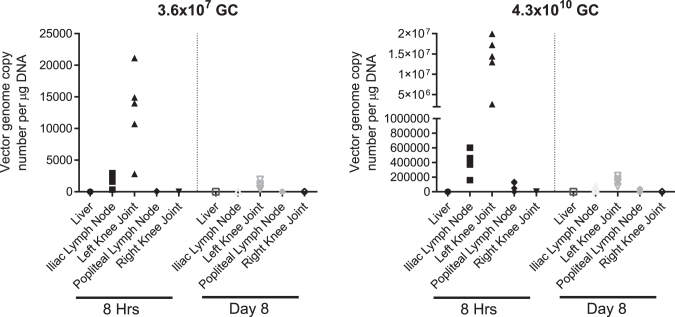
FX201 predominantly remains in the joint following IA administration. Rats were given a single IA injection of either 3.6 × 10^7^ GC or 4.3 × 10^10^ GC FX201 into the *left* hind knee joint. Animals were sacrificed at either 8 h or 8 days after dose and vector genome copy numbers were measured using a validated qPCR method in liver, iliac lymph node, *left* knee joint, popliteal lymph node, and *right* knee joint. qPCR, quantitative polymerase chain reaction.

Rats received a single IA injection of FX201 into the left hind knee. Animals were sacrificed at either 8 h or 8 days after this dose (*n* = 5 per time point) and vector genome copy numbers were measured in the entire injected knee joint as well as the key peripheral organs. Vector genome copy numbers were highest locally in the injected knee joint, with only sporadic, low copy numbers detected in the iliac and popliteal lymph nodes, demonstrating that the vector predominantly remains localized in the knee joint (Fig. 3). A decrease in local knee vector genome copy numbers was observed from 8 h to 8 days after dose, consistent with previous reports, demonstrating a rapid decrease in vector DNA following injection.^29^

### HDAd-ratIL-1Ra was well tolerated following IA injection in rats

The safety profile of HDAd-rat-IL-1Ra was examined in the 3-month Good Laboratory Practice toxicology study in rats. Overall, no treatment-related mortalities (Supplementary Table S4), clinical signs (Supplementary Table S5), or changes in body weight (Supplementary Fig. S4) or food consumption (Supplementary Table S6) were observed during the study. In addition, no adverse changes in hematology (Supplementary Table S7), clinical chemistry (Supplementary Table S8), or urinalysis (Supplementary Table S9) related to treatment and/or ACLT were detected.

Histology was assessed in HDAd-ratIL-1Ra–treated rats on days 29 and 92 after dose. No HDAd-ratIL-1Ra-related microscopic change was detected in any examined organ other than the treated right knee (Supplementary Table S10). On day 29, increased incidence of mononuclear cell infiltration and synovial hypertrophy/hyperplasia was observed in the right knee joint of ACLT-operated rats receiving HDAd-ratIL-1Ra at 4.3 × 10^10^ GC/dose compared with those receiving lower doses of HDAd-ratIL-1Ra, vehicle, or no treatment (Supplementary Table S10). These changes were nonadverse and resolved by day 92. No clinical findings indicative of reduced mobility or pain were observed.

To assess immunogenicity of HDAd-ratIL-1Ra, anti-Ad5 antibody titers were analyzed in serum samples from HDAd-ratIL-1Ra–treated rats on days 29 and 92 after dosing (*n* = 12 per time point). Anti-Ad5 antibody titers increased in a dose-dependent manner following a single IA injection of HDAd-ratIL-1Ra, with higher titers measured on day 29 compared with day 92 (Table 1). Similarly, the number of positive samples following IA injection decreased from days 29 to 92 in rats receiving HDAd-ratIL-1Ra at 3.2 × 10^8^ GC/dose (7 vs. 4 samples).

**Table 1.** Anti-Ad5 antibody titers in ACLT-operated rats receiving HDAd-ratIL-1Ra treatment

	No. of Pre-dose Positive Samples	Day 29 Titer Range (% Positive Samples)	Day 92 Titer Range (% Positive Samples)
ACLT/HDAd-ratIL-1Ra (3.2 × 10^8^ GC/dose)	0/12	1/20 to 1/80 (58.3)	1/20 to 1/40 (33.3)
ACLT/HDAd-ratIL-1Ra (3.1 × 10^9^GC/dose)	0/12	1/20 to 1/640 (91.7)	1/20 to 1/80 (91.7)
ACLT/HDAd-ratIL-1Ra (4.3 × 10^10^ GC/dose)	0/12	1/40 to 1/1280 (100)	1/160 to 1/640 (100)

Anti-Ad5 titers were only analyzed in rats treated with HDAd-ratIL-1Ra.

ACLT, anterior cruciate ligament transection; Ad5, adenovirus serotype 5; GC, genome copies; HDAd, helper-dependent adenovirus.

However, the number of positive samples following IA injection remained unchanged from days 29 to 92 for rats receiving HDAd-ratIL-1Ra at 3.1 × 10^9^ or 4.3 × 10^10^ GC/dose. Taken together, these results suggest that the titer of circulating anti-Ad5 antibodies in HDAd-ratIL-1Ra–treated rats was dose dependent and decreased with time following IA injection.

**Figure 4.**
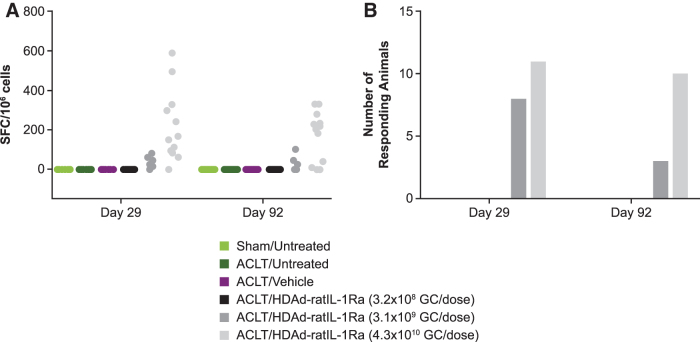
T cell-mediated IFN-γ responses to Ad5 peptides decrease from days 29 to 92 after dose following HDAd-ratIL-1Ra treatment. ACLT surgery was performed on 8- to 9-week-old male rats, and a single IA injection of HDAd-ratIL-1Ra or vehicle was administered 28 days after surgery. To analyze T cell-mediated IFN-γ responses following IA injection of HDAd-ratIL-1Ra, splenocytes were collected from HDAd-ratIL-1Ra–treated rats following sacrifice on days 29 and 92 after dose and stimulated with Ad5 peptides (3.0 nmol/mL). **(A)** IFN-γ-producing splenocytes were quantified as SFC by enzyme-linked immunosorbent spot assay. Robust IFN-γ responses were observed on day 29 following HDAd-ratIL-1Ra treatment; however, responses decreased through day 92. Minimal to no response was observed in sham/untreated, ACLT/untreated, ACLT/vehicle, or ACLT/HDAd-ratIL-1Ra 3.2 × 10^8^ GC/dose groups at both time points. **(B)** The number of rats with IFN-γ-producing splenocytes upon Ad5 peptide stimulation was counted on days 29 and 92 after dose. A significant increase in the number of responding rats was observed in animals dosed with 4.3 × 10^10^ GC/dose compared to sham/untreated, ACLT/untreated, ACLT/vehicle, and ACLT/HDAd-ratIL-1Ra (3.2 × 10^8^ GC/dose) on days 29 and 92 (*p* < 0.05 for each comparison, two-way analysis of variance with Tukey's multiple comparisons test). The number of responding animals decreased from days 29 to 92 after dose for the ACLT/HDAd-ratIL-1Ra 3.2 × 10^9^ GC/dose group. Ad5, adenovirus serotype 5; IFN-γ, interferon-γ; SFC, spot-forming cells.

Finally, T cell responses to Ad5 capsid of the HDAd-ratIL-1Ra vector were assessed by IFN-γ ELISpot assay in rat splenocytes from each study group following *ex vivo* stimulation with Ad5 peptides on days 29 and 92 after dosing (*n* = 12 per time point). Robust dose-dependent induction of Ad5 reactive T cells was observed in HDAd-ratIL-1Ra–treated rats (Fig. 4A). At the highest HDAd-ratIL-1Ra dose (4.3 × 10^10^ GC/dose), IFN-γ responses were sustained from days 29 to 92.

However, IFN-γ responses decreased from days 29 to 92 in splenocytes from rats that received HDAd-ratIL-1Ra at 3.1 × 10^9^ GC/dose, and a minimal IFN-γ response was detected in splenocytes from rats that received HDAd-ratIL-1Ra at 3.2 × 10^8^ GC/dose on days 29 or 92. In addition, the number of rats treated with HDAd-ratIL-1Ra at 3.2 × 10^8^ and 3.1 × 10^9^ GC/dose that exhibited a T cell response to Ad5 peptide stimulation decreased from days 29 to 92 (Fig. 4B). In contrast, no clear decrease in number of rats with T cells reactive to Ad5 was evident at the highest dose level of 4.3 × 10^10^ GC/dose HDAd-ratIL-1Ra.

## DISCUSSION

These GLP and companion biodistribution studies assessed the efficacy, biodistribution, and safety of HDAd-ratIL-1Ra and the biodistribution of FX201. Following a single IA injection, HDAd-ratIL-1Ra mitigated the development of OA-like changes in cartilage, bone, and the synovial membrane 12 weeks following ACLT surgery. In addition, following a single injection of FX201, the vector genomes predominantly remain locally within the injected knee joint, with sporadic, low copy number distribution observed in the draining lymph nodes, liver, lung, and spleen. Finally, HDAd-ratIL-1Ra was well tolerated, and no systemic toxicity was observed throughout the study.

Anti-Ad5 antibody titers and Ad5 peptide-stimulated IFN-γ responses were dose dependent and decreased from days 29 to 92 in rats receiving HDAd-ratIL-1Ra treatment. Taken together, these results suggest that HDAd-ratIL-1Ra alleviates OA-related joint damage in ACLT-operated rats, remains localized to the joint space, and is well tolerated.

Consistent with equine and murine data, efficacy data suggest that HDAd-ratIL-1Ra decreased OA-induced joint damage in ACLT-operated rats, even at a 10-fold lower dose (3.6 × 10^7^ GC/dose), than that tested in mice (1.6 × 10^8^ GC/dose).^24^ The decreases in OARSI scores following IA injection of HDAd-ratIL-1Ra were mainly driven by reduced severity of structural changes in cartilage and chondrocyte loss.

Similarly, biodistribution of HDAd-ratIL-1Ra was comparable to that of HDAd-eqIL-1Ra, the equine surrogate of FX201.^24^ Consistent with the pattern observed in horses, HDAd-ratIL-1Ra and FX201 vector concentrations were highest at the injection site and proximal tissues with little to no detection in other tissues; this pattern persisted for the duration of the 92-day study. Furthermore, biodistribution patterns of FX201 and HDAd-ratIL-1Ra outside the joint were generally reflective of immune cell trafficking to local draining lymph nodes and were thus consistent with the expected immune response to the Ad5 capsid.

Safety findings with HDAd-ratIL-1Ra were comparable to those observed with the equine surrogate of FX201.^24^ Increased incidence of mononuclear cell infiltration and synovial hypertrophy/hyperplasia was observed in rats receiving the highest tested HDAd-ratIL-1Ra dose of 4.3 × 10^10^ GC/dose on day 29 following that dose, reflective of the observed immune response to the vector. However, both resolved by day 92 and were considered nonadverse. Together, these results suggest that HDAd-ratIL-1Ra stimulated a transient adaptive immune response following IA injection in ACLT-operated rats as was observed with HDAd-eqIL-1Ra in horses.

The no observed adverse effect level in our safety study (4.3 × 10^10^ GC/dose) is 1,000-fold greater than the minimal efficacious dose (3.6 × 10^7^ GC/dose) observed. Although an elevated immune response was observed in rats receiving HDAd-ratIL-1Ra at 4.3 × 10^10^ GC/dose on day 29, it should be noted that this dose was 1,000-fold higher than the minimal efficacious dose and exceeded the expected therapeutic range. Therefore, HDAd-ratIL-1Ra has demonstrated a favorable therapeutic index in ACLT-operated rats.

## CONCLUSIONS

These results suggest that HDAd-ratIL-1Ra provides a local disease-modifying effect and demonstrates a favorable safety profile in the ACLT rat model of OA. These results support further development of FX201 as a therapeutic treatment option for knee OA. A Phase 1 study (NCT04119687) to assess the safety and tolerability of FX201 in patients with knee OA is currently underway.

## DATA STATEMENT

The datasets generated and/or analyzed within this publication are available from the corresponding author on reasonable request.

## MEDICAL WRITING AND/OR EDITORIAL ASSISTANCE

Professional medical writing and editorial assistance in the preparation of this article were provided by Stephanie Agbu, PhD, ApotheCom, Yardley, PA.

## Supplementary Material

Supplemental data

Supplemental data

Supplemental data

Supplemental data

Supplemental data

Supplemental data

Supplemental data

Supplemental data

Supplemental data

Supplemental data

Supplemental data

Supplemental data

Supplemental data

Supplemental data

Supplemental data

Supplemental data
