# Supplementary material for: Efficacy and Safety of FX201, a Novel Intra-Articular IL-1Ra Gene Therapy for Osteoarthritis Treatment, in a Rat Model
Source: Hum Gene Ther. 2022 May 16;33(9-10):541–9. doi: 10.1089/hum.2021.131 (PMC9142767; doi:10.1089/hum.2021.131)
Supplement: Supplemental data [file Supp_FigS1.docx]

**Figure S1. Study designs for efficacy, GLP biodistribution, and GLP safety studies of HDAd-ratIL-1Ra.** **(A)** For the efficacy study, 8- to 9-week-old male rats were assigned to one of four study groups and underwent ACLT surgery on day 0. A single IA injection of HDAd-ratIL-1Ra or vehicle was administered 1 week after surgery, and rats were sacrificed at week 11 after dosing for histological analysis. **(B)** For the GLP biodistribution study, 8- to 9-week-old male and female rats were assigned to one of three study groups, underwent ACLT surgery on day -28, and received a single IA injection of HDAd-ratIL-1Ra, FX201, or were left untreated for 28 days after surgery (day 0). Rats were sacrificed at days 8, 29, and 92 after dose (*n* = 12 per time point). **(C)** For the GLP safety study, 8- to 9-week-old male rats were assigned to one of six study groups, underwent ACLT or sham surgery on day 28, and received a single IA injection of HDAd-ratIL-1Ra or vehicle 28 days following surgery (day 0). Rats were sacrificed at days 29 and 92 after dose.

ACLT, anterior cruciate ligament transection; GC, genome copies; GLP, Good Laboratory Practice; HDAd, helper-dependent adenovirus; IA, intra-articular; IL-1Ra, interleukin-1 receptor antagonist.
