# Supplementary material for: Efficacy and Safety of FX201, a Novel Intra-Articular IL-1Ra Gene Therapy for Osteoarthritis Treatment, in a Rat Model
Source: Hum Gene Ther. 2022 May 16;33(9-10):541–9. doi: 10.1089/hum.2021.131 (PMC9142767; doi:10.1089/hum.2021.131)
Supplement: Supplemental data [file Supp_TableS1.docx]

**Table S1. OARSI scoring system used in Figures 1 and 2^26,27^**

| **I. Structural changes (0-10)** |  |
| --- | --- |
| Normal | 0 |
| Surface irregularities (undulating articular surface but no fibrillation) | 1 |
| Minimal mild superficial fibrillation (less than 10% of articular cartilage thickness) < 50% of the plateau/condyle surface | 2 |
| Minimal mild superficial fibrillation (less than 10% of articular cartilage thickness) > 50% of the plateau/condyle surface | 3 |
| Fibrillation/clefts/fissure/loss of articular cartilage involving superficial 1/3 of articular cartilage <50% of the plateau/condyle surface | 4 |
| Fibrillation/clefts/fissure/loss of articular cartilage involving superficial 1/3 of articular cartilage >50% of the plateau/condyle surface | 5 |
| Fibrillation/clefts/fissure/loss of articular cartilage involving superficial 1/3 to 2/3 of articular cartilage <50% of the plateau/condyle surface | 6 |
| Fibrillation/clefts/fissure/loss of articular cartilage involving superficial 1/3 to 2/3 of articular cartilage >50% of the plateau/condyle surface | 7 |
| Fibrillation/clefts/fissure/loss of articular cartilage involving superficial >2/3 of articular cartilage <50% of the plateau/condyle surface | 8 |
| Fibrillation/clefts/fissure/loss of articular cartilage involving superficial >2/3 of articular cartilage >50% of the plateau/condyle surface | 9 |
| Fibrillation/clefts/fissure/loss of articular cartilage to subchondral bone | 10 |
| **II. SOFG staining (0-6)** |  |
| Normal | 0 |
| Loss of staining in superficial zone of articular cartilage involving <50% plateau/condyle | 1 |
| Loss of staining in superficial zone of articular cartilage involving ≥50% plateau/condyle | 2 |
| Loss of staining in upper 2/3 of articular cartilage involving <50% plateau/condyle | 3 |
| Loss of staining in upper 2/3 of articular cartilage involving ≥50% plateau/condyle | 4 |
| Loss of staining in all the articular cartilage involving <50% plateau/condyle | 5 |
| Loss of staining in all the articular cartilage involving >50% plateau/condyle | 6 |
| **III. Clones (cluster) formation (0-3)** |  |
| None | 0 |
| < 4 clones | 1 |
| ≥4 but <8 clones | 2 |
| ≥8 clones | 3 |
| **IV. Loss of chondrocytes (0-6)** |  |
| Normal | 0 |
| Focal chondrocyte loss | 1 |
| Loss of chondrocytes in superficial zone <50% of the condyle/plateau | 2 |
| Loss of chondrocytes in superficial zone >50% of the condyle/plateau | 3 |
| Loss of chondrocytes in mid zone <50% of the condyle/plateau | 4 |
| Loss of chondrocytes in mid zone >50% of the condyle/plateau | 5 |
| Diffuse loss of chondrocytes | 6 |
