# Supplementary material for: Efficacy and Safety of FX201, a Novel Intra-Articular IL-1Ra Gene Therapy for Osteoarthritis Treatment, in a Rat Model
Source: Hum Gene Ther. 2022 May 16;33(9-10):541–9. doi: 10.1089/hum.2021.131 (PMC9142767; doi:10.1089/hum.2021.131)
Supplement: Supplemental data [file Supp_TableS2.docx]

**Table S2. Individual OARSI scores for sham- and ACLT-operated rats at week 11**

| **Sham/untreated** | | | | | | | | | | | | |
| --- | --- | --- | --- | --- | --- | --- | --- | --- | --- | --- | --- | --- |
|  |  |  |  |  |  | | | |  | | | |
| **Animal**  **number** | **Compartment** | **Structural**  **changes** | **SOFG**  **staining loss** | **Clone**  **formation** | **Chondrocyte loss** | | | | **Composite**  **score** | | | |
|  |  |  |  |  |  | | | |  | | | |
| 1001 | FM | 0 | 0 | 0 | 0 | | | | 0 | | | |
|  | FL | 0 | 0 | 0 | 0 | | | | 0 | | | |
|  | TM | 1 | 1 | 0 | 0 | | | | 2 | | | |
|  | TL | 1 | 1 | 0 | 0 | | | | 2 | | | |
|  | Sum | 2 | 2 | 0 | 0 | | | | 4 | | | |
|  |  |  |  |  |  | | | |  | | | |
| 1002 | FM | 2 | 1 | 0 | 0 | | | | 3 | | | |
|  | FL | 2 | 1 | 0 | 0 | | | | 3 | | | |
|  | TM | 2 | 1 | 0 | 1 | | | | 4 | | | |
|  | TL | 1 | 1 | 0 | 0 | | | | 2 | | | |
|  | Sum | 7 | 4 | 0 | 1 | | | | 12 | | | |
|  |  |  |  |  |  | | | |  | | | |
| 1003 | FM | 0 | 0 | 0 | 0 | | | | 0 | | | |
|  | FL | 0 | 0 | 0 | 0 | | | | 0 | | | |
|  | TM | 1 | 1 | 0 | 0 | | | | 2 | | | |
|  | TL | 0 | 1 | 0 | 0 | | | | 1 | | | |
|  | Sum | 1 | 2 | 0 | 0 | | | | 3 | | | |
|  |  |  |  |  |  | | | |  | | | |
| 1004 | FM | 0 | 0 | 0 | 0 | | | | 0 | | | |
|  | FL | 0 | 0 | 0 | 0 | | | | 0 | | | |
|  | TM | 2 | 1 | 0 | 1 | | | | 4 | | | |
|  | TL | 0 | 0 | 0 | 0 | | | | 0 | | | |
|  | Sum | 2 | 1 | 0 | 1 | | | | 4 | | | |
|  |  |  |  |  |  | | | |  | | | |
| 1005 | FM | 0 | 0 | 0 | 0 | | | | 0 | | | |
|  | FL | 1 | 1 | 0 | 0 | | | | 2 | | | |
|  | TM | 1 | 1 | 0 | 0 | | | | 2 | | | |
|  | TL | 1 | 1 | 0 | 0 | | | | 2 | | | |
|  | Sum | 3 | 3 | 0 | 0 | | | | 6 | | | |
| 1006 | FM | 0 | 0 | 0 | 0 | | | | 0 | | | |
|  | FL | 0 | 0 | 0 | 0 | | | | 0 | | | |
|  | TM | 1 | 1 | 0 | 0 | | | | 2 | | | |
|  | TL | 1 | 1 | 0 | 0 | | | | 2 | | | |
|  | Sum | 2 | 2 | 0 | 0 | | | | 4 | | | |
|  |  |  |  |  |  | | | |  | | | |
| 1007 | FM | 0 | 0 | 0 | 0 | | | | 0 | | | |
|  | FL | 0 | 0 | 0 | 0 | | | | 0 | | | |
|  | TM | 2 | 1 | 0 | 1 | | | | 4 | | | |
|  | TL | 0 | 0 | 0 | 0 | | | | 0 | | | |
|  | Sum | 2 | 1 | 0 | 1 | | | | 4 | | | |
|  |  |  |  |  |  | | | |  | | | |
| 1008 | FM | 0 | 0 | 0 | 0 | | | | 0 | | | |
|  | FL | 1 | 1 | 0 | 1 | | | | 3 | | | |
|  | TM | 1 | 1 | 0 | 0 | | | | 2 | | | |
|  | TL | 1 | 1 | 0 | 0 | | | | 2 | | | |
|  | Sum | 3 | 3 | 0 | 1 | | | | 7 | | | |
|  |  |  |  |  |  | | | |  | | | |
| 1009 | FM | 0 | 0 | 0 | 0 | | | | 0 | | | |
|  | FL | 1 | 1 | 0 | 0 | | | | 2 | | | |
|  | TM | 1 | 1 | 0 | 0 | | | | 2 | | | |
|  | TL | 1 | 2 | 0 | 0 | | | | 3 | | | |
|  | Sum | 3 | 4 | 0 | 0 | | | | 7 | | | |
|  |  |  |  |  |  | | | |  | | | |
| 1010 | FM | 0 | 0 | 0 | 0 | | | | 0 | | | |
|  | FL | 1 | 0 | 0 | 0 | | | | 1 | | | |
|  | TM | 1 | 1 | 0 | 0 | | | | 2 | | | |
|  | TL | 1 | 1 | 0 | 0 | | | | 2 | | | |
|  | Sum | 3 | 2 | 0 | 0 | | | | 5 | | | |
| **ACLT/vehicle** | | | | | | | | | | | |  |
| **Animal**  **number** | **Compartment** | **Structural**  **changes** | **SOFG**  **staining loss** | **Clone**  **formation** | **Chondrocyte loss** | | | **Composite**  **score** | | | |  |
|  |  |  |  |  |  | | |  | | | |  |
| 2001 | FM | 4 | 2 | 0 | 1 | | | 7 | | | |  |
|  | FL | 1 | 0 | 0 | 0 | | | 1 | | | |  |
|  | TM | 1 | 2 | 0 | 0 | | | 3 | | | |  |
|  | TL | 0 | 0 | 0 | 0 | | | 0 | | | |  |
|  | Sum | 6 | 4 | 0 | 1 | | | 11 | | | |  |
|  |  |  |  |  |  | | |  | | | |  |
| 2002 | FM | 0 | 0 | 0 | 0 | | | 0 | | | |  |
|  | FL | 0 | 1 | 0 | 1 | | | 2 | | | |  |
|  | TM | 1 | 1 | 0 | 0 | | | 2 | | | |  |
|  | TL | 1 | 1 | 0 | 0 | | | 2 | | | |  |
|  | Sum | 2 | 3 | 0 | 1 | | | 6 | | | |  |
|  |  |  |  |  |  | | |  | | | |  |
| 2003 | FM | 4 | 5 | 3 | 4 | | | 16 | | | |  |
|  | FL | 1 | 2 | 0 | 0 | | | 3 | | | |  |
|  | TM | 3 | 2 | 0 | 2 | | | 7 | | | |  |
|  | TL | 10 | 5 | 1 | 4 | | | 20 | | | |  |
|  | Sum | 18 | 14 | 4 | 10 | | | 46 | | | |  |
|  |  |  |  |  |  | | |  | | | |  |
| 2004 | FM | 2 | 1 | 0 | 1 | | | 4 | | | |  |
|  | FL | 1 | 1 | 0 | 0 | | | 2 | | | |  |
|  | TM | 5 | 2 | 0 | 3 | | | 10 | | | |  |
|  | TL | 2 | 1 | 0 | 0 | | | 3 | | | |  |
|  | Sum | 10 | 5 | 0 | 4 | | | 19 | | | |  |
|  |  |  |  |  |  | | |  | | | |  |
| 2005 | FM | 6 | 5 | 1 | 4 | | | 16 | | | |  |
|  | FL | 1 | 1 | 0 | 0 | | | 2 | | | |  |
|  | TM | 1 | 2 | 0 | 0 | | | 3 | | | |  |
|  | TL | 2 | 2 | 0 | 1 | | | 5 | | | |  |
|  | Sum | 10 | 10 | 1 | 5 | | | 26 | | | |  |
|  |  |  |  |  |  | | |  | | | |  |
|  |  |  |  |  |  | | |  | | | |  |
| 2006 | FM | 1 | 0 | 0 | 1 | | | 2 | | | |  |
|  | FL | 0 | 0 | 0 | 0 | | | 0 | | | |  |
|  | TM | 2 | 2 | 0 | 0 | | | 4 | | | |  |
|  | TL | 2 | 2 | 0 | 0 | | | 4 | | | |  |
|  | Sum | 5 | 4 | 0 | 1 | | | 10 | | | |  |
|  |  |  |  |  |  | | |  | | | |  |
| 2007 | FM | 1 | 1 | 0 | 0 | | | 2 | | | |  |
|  | FL | 1 | 1 | 0 | 0 | | | 2 | | | |  |
|  | TM | 2 | 1 | 0 | 0 | | | 3 | | | |  |
|  | TL | 1 | 1 | 0 | 0 | | | 2 | | | |  |
|  | Sum | 5 | 4 | 0 | 0 | | | 9 | | | |  |
|  |  |  |  |  |  | | |  | | | |  |
| 2008 | FM | 4 | 2 | 1 | 2 | | | 9 | | | |  |
|  | FL | 1 | 1 | 0 | 1 | | | 3 | | | |  |
|  | TM | 1 | 1 | 0 | 0 | | | 2 | | | |  |
|  | TL | 1 | 2 | 0 | 1 | | | 4 | | | |  |
|  | Sum | 7 | 6 | 1 | 4 | | | 18 | | | |  |
|  |  |  |  |  |  | | |  | | | |  |
| 2009 | FM | 2 | 1 | 0 | 0 | | | 3 | | | |  |
|  | FL | 1 | 1 | 0 | 1 | | | 3 | | | |  |
|  | TM | 10 | 3 | 0 | 4 | | | 17 | | | |  |
|  | TL | 2 | 2 | 0 | 0 | | | 4 | | | |  |
|  | Sum | 15 | 7 | 0 | 5 | | | 27 | | | |  |
|  |  |  |  |  |  | | |  | | | |  |
| 2010 | FM | 3 | 2 | 0 | 0 | | | 5 | | | |  |
|  | FL | 1 | 2 | 0 | 0 | | | 3 | | | |  |
|  | TM | 4 | 2 | 0 | 2 | | | 8 | | | |  |
|  | TL | 2 | 2 | 0 | 0 | | | 4 | | | |  |
|  | Sum | 10 | 8 | 0 | 2 | | | 20 | | | |  |
|  |  |  |  |  |  | | |  | | | |  |
|  |  |  |  |  |  | | |  | | | |  |
|  |  |  |  |  |  | | |  | | | |  |
| 2011 | FM | 1 | 2 | 0 | 1 | | | 4 | | | |  |
|  | FL | 1 | 1 | 0 | 0 | | | 2 | | | |  |
|  | TM | 3 | 2 | 0 | 1 | | | 6 | | | |  |
|  | TL | 2 | 2 | 0 | 0 | | | 4 | | | |  |
|  | Sum | 7 | 7 | 0 | 2 | | | 16 | | | |  |
|  |  |  |  |  |  | | |  | | | |  |
| 2012 | FM | 2 | 1 | 0 | 0 | | | 3 | | | |  |
|  | FL | 2 | 2 | 1 | 0 | | | 5 | | | |  |
|  | TM | 3 | 2 | 0 | 2 | | | 7 | | | |  |
|  | TL | 2 | 1 | 0 | 1 | | | 4 | | | |  |
|  | Sum | 9 | 6 | 1 | 3 | | | 19 | | | |  |
| **ACLT/HDAd-ratIL-1Ra (3.6**×**10^7^ GC/dose)** | | | | | | | | | | |  |  |
| **Animal**  **number** | **Compartment** | **Structural**  **changes** | **SOFG**  **staining loss** | **Clone**  **formation** | **Chondrocyte loss** | | **Composite**  **score** | | | |  |  |
|  |  |  |  |  |  | |  | | | |  |  |
| 3001 | FM | 4 | 1 | 0 | 1 | | 6 | | | |  |  |
|  | FL | 0 | 0 | 0 | 0 | | 0 | | | |  |  |
|  | TM | 2 | 2 | 0 | 0 | | 4 | | | |  |  |
|  | TL | 0 | 1 | 0 | 0 | | 1 | | | |  |  |
|  | Sum | 6 | 4 | 0 | 1 | | 11 | | | |  |  |
|  |  |  |  |  |  | |  | | | |  |  |
| 3002 | FM | 1 | 1 | 0 | 0 | | 2 | | | |  |  |
|  | FL | 0 | 0 | 0 | 0 | | 0 | | | |  |  |
|  | TM | 1 | 1 | 0 | 0 | | 2 | | | |  |  |
|  | TL | 1 | 1 | 0 | 0 | | 2 | | | |  |  |
|  | Sum | 3 | 3 | 0 | 0 | | 6 | | | |  |  |
|  |  |  |  |  |  | |  | | | |  |  |
| 3003 | FM | 0 | 0 | 0 | 0 | | 0 | | | |  |  |
|  | FL | 1 | 2 | 0 | 1 | | 4 | | | |  |  |
|  | TM | 2 | 2 | 0 | 0 | | 4 | | | |  |  |
|  | TL | 1 | 1 | 0 | 0 | | 2 | | | |  |  |
|  | Sum | 4 | 5 | 0 | 1 | | 10 | | | |  |  |
|  |  |  |  |  |  | |  | | | |  |  |
| 3004 | FM | 1 | 0 | 0 | 0 | | 1 | | | |  |  |
|  | FL | 0 | 0 | 0 | 0 | | 0 | | | |  |  |
|  | TM | 2 | 2 | 0 | 0 | | 4 | | | |  |  |
|  | TL | 1 | 1 | 0 | 0 | | 2 | | | |  |  |
|  | Sum | 4 | 3 | 0 | 0 | | 7 | | | |  |  |
|  |  |  |  |  |  | |  | | | |  |  |
| 3005 | FM | 1 | 1 | 0 | 1 | | 3 | | | |  |  |
|  | FL | 1 | 1 | 0 | 0 | | 2 | | | |  |  |
|  | TM | 1 | 1 | 0 | 0 | | 2 | | | |  |  |
|  | TL | 2 | 2 | 0 | 1 | | 5 | | | |  |  |
|  | Sum | 5 | 5 | 0 | 2 | | 12 | | | |  |  |
|  |  |  |  |  |  | |  | | | |  |  |
| 3006 | FM | 10 | 5 | 3 | 6 | | 24 | | | |  |  |
|  | FL | 1 | 1 | 0 | 0 | | 2 | | | |  |  |
|  | TM | 5 | 2 | 0 | 3 | | 10 | | | |  |  |
|  | TL | 1 | 1 | 0 | 0 | | 2 | | | |  |  |
|  | Sum | 17 | 9 | 3 | 9 | | 38 | | | |  |  |
|  |  |  |  |  |  | |  | | | |  |  |
| 3007 | FM | 0 | 1 | 0 | 0 | | 1 | | | |  |  |
|  | FL | 0 | 1 | 0 | 1 | | 2 | | | |  |  |
|  | TM | 4 | 1 | 0 | 2 | | 7 | | | |  |  |
|  | TL | 2 | 1 | 0 | 0 | | 3 | | | |  |  |
|  | Sum | 6 | 4 | 0 | 3 | | 13 | | | |  |  |
|  |  |  |  |  |  | |  | | | |  |  |
| 3008 | FM | 0 | 5 | 0 | 1 | | 6 | | | |  |  |
|  | FL | 0 | 1 | 0 | 1 | | 2 | | | |  |  |
|  | TM | 1 | 2 | 0 | 0 | | 3 | | | |  |  |
|  | TL | 1 | 2 | 0 | 0 | | 3 | | | |  |  |
|  | Sum | 2 | 10 | 0 | 2 | | 14 | | | |  |  |
|  |  |  |  |  |  | |  | | | |  |  |
| 3009 | FM | 8 | 5 | 2 | 6 | | 21 | | | |  |  |
|  | FL | 0 | 1 | 0 | 0 | | 1 | | | |  |  |
|  | TM | 10 | 5 | 1 | 6 | | 22 | | | |  |  |
|  | TL | 2 | 2 | 0 | 0 | | 4 | | | |  |  |
|  | Sum | 20 | 13 | 3 | 12 | | 48 | | | |  |  |
|  |  |  |  |  |  | |  | | | |  |  |
| 3010 | FM | 1 | 1 | 0 | 0 | | 2 | | | |  |  |
|  | FL | 1 | 1 | 0 | 0 | | 2 | | | |  |  |
|  | TM | 2 | 2 | 0 | 0 | | 4 | | | |  |  |
|  | TL | 1 | 1 | 0 | 0 | | 2 | | | |  |  |
|  | Sum | 5 | 5 | 0 | 0 | | 10 | | | |  |  |
| 3011 | FM | 0 | 3 | 2 | 1 | | 6 | | | |  |  |
|  | FL | 0 | 0 | 0 | 0 | | 0 | | | |  |  |
|  | TM | 3 | 2 | 0 | 1 | | 6 | | | |  |  |
|  | TL | 1 | 2 | 0 | 0 | | 3 | | | |  |  |
|  | Sum | 4 | 7 | 2 | 2 | | 15 | | | |  |  |
|  |  |  |  |  |  | |  | | | |  |  |
| 3012 | FM | 1 | 1 | 0 | 0 | | 2 | | | |  |  |
|  | FL | 0 | 0 | 0 | 0 | | 0 | | | |  |  |
|  | TM | 3 | 2 | 0 | 0 | | 5 | | | |  |  |
|  | TL | 1 | 1 | 0 | 0 | | 2 | | | |  |  |
|  | Sum | 5 | 4 | 0 | 0 | | 9 | | | |  |  |
| **ACLT/HDAd-ratIL-1Ra (3.1**×**10^8^ GC/dose)** | | | | | | | | | |  |  |  |
| **Animal**  **number** | **Compartment** | **Structural**  **changes** | **SOFG**  **staining loss** | **Clone**  **formation** | **Chondrocyte loss** | **Composite**  **score** | | | |  |  |  |
|  |  |  |  |  |  |  | | | |  |  |  |
| 4001 | FM | 1 | 2 | 0 | 1 | 4 | | | |  |  |  |
|  | FL | 0 | 0 | 0 | 0 | 0 | | | |  |  |  |
|  | TM | 0 | 2 | 0 | 0 | 2 | | | |  |  |  |
|  | TL | 0 | 1 | 0 | 0 | 1 | | | |  |  |  |
|  | Sum | 1 | 5 | 0 | 1 | 7 | | | |  |  |  |
|  |  |  |  |  |  |  | | | |  |  |  |
| 4002 | FM | 1 | 2 | 0 | 0 | 3 | | | |  |  |  |
|  | FL | 0 | 0 | 0 | 0 | 0 | | | |  |  |  |
|  | TM | 1 | 1 | 0 | 0 | 2 | | | |  |  |  |
|  | TL | 1 | 2 | 0 | 0 | 3 | | | |  |  |  |
|  | Sum | 3 | 5 | 0 | 0 | 8 | | | |  |  |  |
|  |  |  |  |  |  |  | | | |  |  |  |
| 4003 | FM | 1 | 1 | 1 | 0 | 3 | | | |  |  |  |
|  | FL | 0 | 0 | 0 | 0 | 0 | | | |  |  |  |
|  | TM | 1 | 2 | 0 | 0 | 3 | | | |  |  |  |
|  | TL | 1 | 2 | 0 | 0 | 3 | | | |  |  |  |
|  | Sum | 3 | 5 | 1 | 0 | 9 | | | |  |  |  |
|  |  |  |  |  |  |  | | | |  |  |  |
| 4004 | FM | 1 | 1 | 0 | 0 | 2 | | | |  |  |  |
|  | FL | 2 | 1 | 0 | 1 | 4 | | | |  |  |  |
|  | TM | 1 | 1 | 0 | 0 | 2 | | | |  |  |  |
|  | TL | 0 | 0 | 0 | 0 | 0 | | | |  |  |  |
|  | Sum | 4 | 3 | 0 | 1 | 8 | | | |  |  |  |
|  |  |  |  |  |  |  | | | |  |  |  |
| 4005 | FM | 1 | 1 | 0 | 0 | 2 | | | |  |  |  |
|  | FL | 2 | 1 | 0 | 0 | 3 | | | |  |  |  |
|  | TM | 1 | 1 | 0 | 0 | 2 | | | |  |  |  |
|  | TL | 1 | 1 | 0 | 0 | 2 | | | |  |  |  |
|  | Sum | 5 | 4 | 0 | 0 | 9 | | | |  |  |  |
|  |  |  |  |  |  |  | | | |  |  |  |
|  |  |  |  |  |  |  | | | |  |  |  |
| 4006 | FM | 1 | 1 | 0 | 0 | 2 | | | |  |  |  |
|  | FL | 1 | 1 | 0 | 0 | 2 | | | |  |  |  |
|  | TM | 2 | 1 | 0 | 1 | 4 | | | |  |  |  |
|  | TL | 1 | 1 | 0 | 0 | 2 | | | |  |  |  |
|  | Sum | 5 | 4 | 0 | 1 | 10 | | | |  |  |  |
|  |  |  |  |  |  |  | | | |  |  |  |
| 4007 | FM | 0 | 0 | 0 | 0 | 0 | | | |  |  |  |
|  | FL | 1 | 1 | 0 | 0 | 2 | | | |  |  |  |
|  | TM | 2 | 1 | 0 | 0 | 3 | | | |  |  |  |
|  | TL | 1 | 1 | 0 | 0 | 2 | | | |  |  |  |
|  | Sum | 4 | 3 | 0 | 0 | 7 | | | |  |  |  |
|  |  |  |  |  |  |  | | | |  |  |  |
| 4008 | FM | 1 | 1 | 0 | 0 | 2 | | | |  |  |  |
|  | FL | 1 | 1 | 0 | 0 | 2 | | | |  |  |  |
|  | TM | 2 | 2 | 0 | 0 | 4 | | | |  |  |  |
|  | TL | 2 | 2 | 0 | 0 | 4 | | | |  |  |  |
|  | Sum | 6 | 6 | 0 | 0 | 12 | | | |  |  |  |
|  |  |  |  |  |  |  | | | |  |  |  |
| 4009 | FM | 2 | 1 | 0 | 0 | 3 | | | |  |  |  |
|  | FL | 2 | 1 | 0 | 1 | 4 | | | |  |  |  |
|  | TM | 1 | 1 | 0 | 0 | 2 | | | |  |  |  |
|  | TL | 2 | 2 | 0 | 0 | 4 | | | |  |  |  |
|  | Sum | 7 | 5 | 0 | 1 | 13 | | | |  |  |  |
|  |  |  |  |  |  |  | | | |  |  |  |
| 4010 | FM | 2 | 2 | 0 | 0 | 4 | | | |  |  |  |
|  | FL | 1 | 1 | 0 | 1 | 3 | | | |  |  |  |
|  | TM | 2 | 2 | 0 | 0 | 4 | | | |  |  |  |
|  | TL | 1 | 1 | 0 | 0 | 2 | | | |  |  |  |
|  | Sum | 6 | 6 | 0 | 1 | 13 | | | |  |  |  |
|  |  |  |  |  |  |  | | | |  |  |  |
| 4011 | FM | 1 | 1 | 0 | 0 | 2 | | | |  |  |  |
|  | FL | 1 | 1 | 0 | 0 | 2 | | | |  |  |  |
|  | TM | 1 | 2 | 0 | 0 | 3 | | | |  |  |  |
|  | TL | 1 | 1 | 0 | 0 | 2 | | | |  |  |  |
|  | Sum | 4 | 5 | 0 | 0 | 9 | | | |  |  |  |
|  |  |  |  |  |  |  | | | |  |  |  |
| 4012 | FM | 0 | 0 | 0 | 0 | 0 | | | |  |  |  |
|  | FL | 0 | 0 | 0 | 0 | 0 | | | |  |  |  |
|  | TM | 0 | 0 | 0 | 0 | 0 | | | |  |  |  |
|  | TL | 1 | 1 | 0 | 0 | 2 | | | |  |  |  |
|  | Sum | 1 | 1 | 0 | 0 | 2 | | | |  |  |  |

ACLT, anterior cruciate ligament transection; FL, lateral femur; FM, medial femur; GC, genome copies; HDAd, helper-dependent adenovirus; IL-1Ra, interleukin-1 receptor antagonist; OARSI, Osteoarthritis Research Society International; TL, lateral tibia; TM, medial tibia; SOFG, Safranin-O/Fast Green.
