# Supplementary material for: Efficacy and Safety of FX201, a Novel Intra-Articular IL-1Ra Gene Therapy for Osteoarthritis Treatment, in a Rat Model
Source: Hum Gene Ther. 2022 May 16;33(9-10):541–9. doi: 10.1089/hum.2021.131 (PMC9142767; doi:10.1089/hum.2021.131)
Supplement: Supplemental data [file Supp_FigS3.docx]

**Figure S3. FX201 minimally distributes systemically following IA administration.** ACLT surgery was performed on 8- to 9-week-old male rats, and a single IA injection of 4.3x10^10^ genome copies of FX201 was administered 28 days after surgery, with animals sacrificed at either **(A)** day 8, **(B)** 29, or **(C)** 92 following injection. Vector biodistribution was evaluated using a validated qPCR method. A similar profile was obtained with HDAd-ratIL-1Ra.
