# Supplementary material for: Efficacy and Safety of FX201, a Novel Intra-Articular IL-1Ra Gene Therapy for Osteoarthritis Treatment, in a Rat Model
Source: Hum Gene Ther. 2022 May 16;33(9-10):541–9. doi: 10.1089/hum.2021.131 (PMC9142767; doi:10.1089/hum.2021.131)
Supplement: Supplemental data [file Supp_TableS5.docx]

**Table S5. Summary of treatment-related clinical observations**

|  | **Males** | | | | | |
| --- | --- | --- | --- | --- | --- | --- |
| Group | 1 | 2 | 3 | 4 | 5 | 6 |
| Dose (GC/dose) | 0 | 0 | 0 | 3.2 x 10^8^ | 3.1 x 10^9^ | 4.3 x 10^10^ |
| Animals per group, *n* | 12 | 12 | 12 | 12 | 12 | 12 |
| Treatment-related clinical observations^a^ | 0 | 0 | 0 | 0 | 0 | 0 |

^a^All clinical observations that occurred were considered unrelated to HDAd-ratIL-1Ra administration because the observations were transient, observed in control animals, procedure related (ACLT and/or intra-articular injection), and/or are commonly observed in this strain and at this age.

GC, genome copies.
