# Supplementary material for: Efficacy and Safety of FX201, a Novel Intra-Articular IL-1Ra Gene Therapy for Osteoarthritis Treatment, in a Rat Model
Source: Hum Gene Ther. 2022 May 16;33(9-10):541–9. doi: 10.1089/hum.2021.131 (PMC9142767; doi:10.1089/hum.2021.131)
Supplement: Supplemental data [file Supp_FigS4.docx]

**Figure S4. Summary of body weights per group over the course of the 92-day toxicology study.** Male rats, 8 to 9 weeks old, were assigned to one of six study groups, underwent ACLT or sham surgery on day 28, and received a single IA injection of HDAd-ratIL-1Ra or vehicle 28 days following surgery (day 0). Body weights were measured for each individual animal throughout the duration of the study and the average body weight in grams was calculated for each treatment group.
