# Supplementary material for: Efficacy and Safety of FX201, a Novel Intra-Articular IL-1Ra Gene Therapy for Osteoarthritis Treatment, in a Rat Model
Source: Hum Gene Ther. 2022 May 16;33(9-10):541–9. doi: 10.1089/hum.2021.131 (PMC9142767; doi:10.1089/hum.2021.131)
Supplement: Supplemental data [file Supp_TableS6.docx]

**Table S6. Summary of Food Consumption by Group**

|  | **Males** | | | | | |
| --- | --- | --- | --- | --- | --- | --- |
| Group | 1 | 2 | 3 | 4 | 5 | 6 |
| Dose (GC/dose) | 0 | 0 | 0 | 3.2 x 10^8^ | 3.1 x 10^9^ | 4.3 x 10^10^ |
| Animals per group, *n* | 12 | 12 | 12 | 12 | 12 | 12 |
| Day -27 to -20 (average grams/day)^a^ | 28.6 | 27.5 | 27.7 | 26.8 | 36.6 | 27.4 |
| Day -20 to -13 | 32.6 | 31.5 | 31.8 | 31.6 | 31.1 | 32.4 |
| Day -13 to -6 | 30.8 | 30.1 | 30.3 | 30.1 | 30.0 | 31.1 |
| Day -6 to 2 | 34.5 | 34.4 | 33.5 | 33.5 | 33.7 | 34.8 |
| Day 2 to 9 | 35.5 | 35.0 | 34.4 | 34.2 | 33.8 | 34.5 |
| Day 9 to 16 | 35.1 | 34.9 | 34.3 | 34.3 | 34.4 | 33.8 |
| Day 16 to 23 | 36.1 | 35.7 | 35.3 | 34.8 | 34.1 | 35.6 |
| Day 23 to 30 | 37.7 | 36.9 | 35.4 | 36.6 | 36.4 | 36.4 |
| Day 30 to 37 | 36.8 | 34.9 | 34.8 | 35.3 | 35.0 | 34.7 |
| Day 37 to 44 | 36.1 | 34.2 | 35.6 | 34.9 | 34.6 | 34.5 |
| Day 44 to 51 | 35.4 | 34.1 | 35.2 | 35.2 | 34.2 | 34.0 |
| Day 51 to 58 | 36.6 | 35.2 | 36.4 | 35.4 | 34.8 | 35.0 |
| Day 58 to 65 | 35.7 | 35.8 | 36.8 | 34.9 | 33.9 | 34.7 |
| Day 65 to 72 | 35.4 | 35.4 | 36.9 | 34.8 | 34.6 | 34.9 |
| Day 72 to 79 | 36.4 | 34.9 | 35.5 | 35.2 | 34.6 | 35.5 |
| Day 79 to 86 | 37.2 | 35.7 | 36.6 | 35.8 | 34.6 | 35.3 |
| Day 86 to 91 | 35.6 | 35.3 | 35.5 | 35.2 | 34.4 | 34.6 |

^a^Average grams of food consumed per group per day for the listed time period.
