# Supplementary material for: Efficacy and Safety of FX201, a Novel Intra-Articular IL-1Ra Gene Therapy for Osteoarthritis Treatment, in a Rat Model
Source: Hum Gene Ther. 2022 May 16;33(9-10):541–9. doi: 10.1089/hum.2021.131 (PMC9142767; doi:10.1089/hum.2021.131)
Supplement: Supplemental data [file Supp_TableS7.docx]

**Table S7. Summary of hematology values**

|  | **Males** | | | | | |
| --- | --- | --- | --- | --- | --- | --- |
| Group | 1 | 2 | 3 | 4 | 5 | 6 |
| Dose (GC/dose) | 0 | 0 | 0 | 3.2 x 10^8^ | 3.1 x 10^9^ | 4.3 x 10^10^ |
| Animals per group, *n* | 12 | 12 | 12 | 12 | 12 | 12 |
| **Day 29** |  |  |  |  |  |  |
| White blood cells (10^3^/µL) | 10.078 | 10.062 | 10.536 | 11.365 | 12.233 | 11.511 |
| Neutrophils (10^3^/µL) | 1.531 | 1.529 | 1.523 | 1.803 | 1.732 | 1.654 |
| Lymphocytes (10^3^/µL) | 7.987 | 7.981 | 8.411 | 8.935 | 9.686 | 9.174 |
| Mononuclear cells (10^3^/µL) | 0.314 | 0.328 | 0.357 | 0.362 | 0.455 | 0.414 |
| Eosinophils (10^3^/µL) | 0.113 | 0.101 | 0.119 | 0.115 | 0.150 | 0.125 |
| Basophils (10^3^/µL) | 0.003 | 0.037 | 0.038 | 0.041 | 0.048 | 0.039 |
| Large unstained cells (10^3^/µL) | 0.103 | 0.087 | 0.091 | 0.107 | 0.157 | 0.104 |
| Red blood cells (10^6^/µL) | 7.818 | 7.828 | 7.987 | 8.122 | 8.043 | 7.911 |
| Hemoglobin (g/dL) | 13.69 | 13.84 | 14.16 | 14.17 | 14.15 | 14.09 |
| Hematocrit (%) | 41.78 | 42.00 | 43.03 | 43.15 | 42.95 | 42.82 |
| Mean corpuscular volume (fL) | 53.44 | 53.62 | 53.93 | 53.17 | 53.42 | 54.17 |
| Mean corpuscular hemoglobin (pg) | 17.49 | 17.69 | 17.74 | 17.46 | 17.61 | 17.85 |
| Mean corpuscular hemoglobin conc. (g/dL) | 32.77 | 33.01 | 32.94 | 32.86 | 32.96 | 32.97 |
| Red blood cell distribution width (%) | 13.20 | 13.84 | 13.08 | 13.34 | 13.30 | 13.62 |
| Platelet count (10^3^/µL) | 1068.4 | 1138.8 | 1093.1 | 1158.7 | 1087.6 | 1155.9 |
| Reticulocytes (10^9^/µL) | 209.38 | 225.08 | 199.68 | 210.37 | 208.63 | 220.18 |
| **Day 92** |  |  |  |  |  |  |
| White blood cells (10^3^/µL) | 7.783 | 8.188 | 8.251 | 8.957 | 8.257 | 7.754 |
| Neutrophils (10^3^/µL) | 1.684 | 1.588 | 1.497 | 1.811 | 1.470 | 1.514 |
| Lymphocytes (10^3^/µL) | 5.591 | 5.988 | 6.237 | 6.574 | 6.239 | 5.697 |
| Mononuclear cells (10^3^/µL) | 0.307 | 0.361 | 0.305 | 0.353 | 0.324 | 0.303 |
| Eosinophils (10^3^/µL) | 0.119 | 0.135 | 0.114 | 0.128 | 0.124 | 0.119 |
| Basophils (10^3^/µL) | 0.017 | 0.022 | 0.018 | 0.021 | 0.021 | 0.018 |
| Large unstained cells (10^3^/µL) | 0.070 | 0.090 | 0.081 | 0.069 | 0.078 | 0.100 |
| Red blood cells (10^6^/µL) | 8.018 | 8.470 | 8.191 | 8.194 | 8.077 | 8.110 |
| Hemoglobin (g/dL) | 13.65 | 14.30 | 13.95 | 14.09 | 13.89 | 13.89 |
| Hematocrit (%) | 41.75 | 44.00 | 42.96 | 43.19 | 42.93 | 42.84 |
| Mean corpuscular volume (fL) | 52.18 | 51.98 | 52.45 | 52.72 | 53.21 | 52.86 |
| Mean corpuscular hemoglobin (pg) | 17.03 | 16.91 | 17.05 | 17.22 | 17.22 | 17.14 |
| Mean corpuscular hemoglobin conc. (g/dL) | 32.66 | 32.52 | 32.49 | 32.66 | 32.37 | 32.43 |
| Red blood cell distribution width (%) | 14.86 | 13.74 | 14.17 | 13.88 | 14.14 | 14.06 |
| Platelet count (10^3^/µL) | 1105.0 | 1079.0 | 1112.8 | 1119.5 | 1103.9 | 1085.7 |
| Reticulocytes (10^9^/µL) | 265.06 | 191.83 | 213.47 | 205.49 | 210.47 | 204.83 |

Mean values per group per timepoint.
