# Supplementary material for: Efficacy and Safety of FX201, a Novel Intra-Articular IL-1Ra Gene Therapy for Osteoarthritis Treatment, in a Rat Model
Source: Hum Gene Ther. 2022 May 16;33(9-10):541–9. doi: 10.1089/hum.2021.131 (PMC9142767; doi:10.1089/hum.2021.131)
Supplement: Supplemental data [file Supp_TableS8.docx]

**Table S8. Summary of clinical chemistry values**

|  | **Males** | | | | | |
| --- | --- | --- | --- | --- | --- | --- |
| Group | 1 | 2 | 3 | 4 | 5 | 6 |
| Dose (GC/dose) | 0 | 0 | 0 | 3.2 x 10^8^ | 3.1 x 10^9^ | 4.3 x 10^10^ |
| Animals per group, *n* | 12 | 12 | 12 | 12 | 12 | 12 |
| **Day 29** |  |  |  |  |  |  |
| Aspartate aminotransferase (U/L) | 123.3 | 116.5 | 99.4 | 105.2 | 93.2 | 92.9 |
| Alanine aminotransferase (U/L) | 34.8 | 37.5 | 31.3 | 36.6 | 32.0 | 30.8 |
| Alkaline phosphatase (U/L) | 105.7 | 112.3 | 98.3 | 99.2 | 99.3 | 99.1 |
| GAMMA-glutamyl transferase (U/L) | 2.0 | 2.0 | 2.0 | 2.0 | 2.0 | 2.0 |
| Creatine kinase (U/L) | 772.4 | 682.8 | 480.1 | 564.0 | 423.9 | 451.2 |
| Total bilirubin (mg/dL) | 0.050 | 0.059 | 0.059 | 0.063 | 0.077 | 0.065 |
| Urea nitrogen (mg/dL) | 13.9 | 13.3 | 13.6 | 12.4 | 13.4 | 12.9 |
| Creatinine (mg/dL) | 0.42 | 0.41 | 0.39 | 0.38 | 0.41 | 0.38 |
| Glucose (mg/dL) | 225.1 | 213.3 | 237.2 | 221.3 | 249.4 | 240.7 |
| Cholesterol (mg/dL) | 54.7 | 68.8 | 67.3 | 63.1 | 59.9 | 58.7 |
| Triglycerides (mg/dL) | 65.8 | 64.7 | 82.8 | 77.3 | 75.4 | 84.7 |
| Total protein (g/dL) | 5.91 | 5.94 | 5.87 | 5.87 | 5.38 | 5.88 |
| Albumin (g/dL) | 3.98 | 3.97 | 3.93 | 3.91 | 3.89 | 3.82 |
| Globulin (g/dL) | 1.93 | 1.98 | 1.94 | 1.96 | 1.94 | 2.07 |
| Albumin/globulin ratio | 2.13 | 2.03 | 2.07 | 2.03 | 2.03 | 1.86 |
| Calcium (mg/dL) | 9.0 | 9.1 | 9.3 | 9.2 | 9.2 | 9.2 |
| Phosphorus (mg/dL) | 6.54 | 7.03 | 7.20 | 7.09 | 7.18 | 7.38 |
| Sodium (mmol/L) | 140.8 | 140.6 | 141.0 | 140.7 | 141.3 | 141.3 |
| Potassium (mmol/L) | 5.29 | 5.14 | 5.21 | 5.38 | 5.20 | 5.33 |
| Chloride (mmol/L) | 101.0 | 100.3 | 101.1 | 101.3 | 101.8 | 102.1 |
| **Day 92** |  |  |  |  |  |  |
| Aspartate aminotransferase (U/L) | 119.4 | 108.9 | 108.0 | 96.1 | 108.3 | 97.5 |
| Alanine aminotransferase (U/L) | 35.1 | 32.6 | 31.8 | 33.7 | 35.1 | 35.4 |
| Alkaline phosphatase (U/L) | 66.4 | 63.6 | 68.5 | 65.7 | 62.6 | 71.7 |
| GAMMA-glutamyl transferase (U/L) | 2.0 | 2.0 | 2.0 | 2.0 | 2.0 | 2.0 |
| Creatine kinase (U/L) | 648.8 | 592.7 | 539.4 | 357.4 | 383.7 | 439.3 |
| Total bilirubin (mg/dL) | 0.088 | 0.072 | 0.080 | 0.087 | 0.087 | 0.069 |
| Urea nitrogen (mg/dL) | 12.5 | 12.4 | 13.0 | 12.1 | 12.9 | 11.7 |
| Creatinine (mg/dL) | 0.34 | 0.34 | 0.35 | 0.33 | 0.36 | 0.32 |
| Glucose (mg/dL) | 181.0 | 177.7 | 200.4 | 194.7 | 203.9 | 205.2 |
| Cholesterol (mg/dL) | 77.7 | 77.8 | 82.3 | 71.4 | 78.3 | 71.3 |
| Triglycerides (mg/dL) | 129.3 | 114.8 | 118.7 | 114.6 | 93.8 | 104.7 |
| Total protein (g/dL) | 6.19 | 6.18 | 6.15 | 6.13 | 6.01 | 6.13 |
| Albumin (g/dL) | 3.97 | 3.83 | 3.87 | 3.88 | 3.81 | 3.85 |
| Globulin (g/dL) | 2.23 | 2.36 | 2.27 | 2.25 | 2.20 | 2.28 |
| Albumin/globulin ratio | 1.82 | 1.63 | 1.73 | 1.75 | 1.78 | 1.71 |
| Calcium (mg/dL) | 9.1 | 9.2 | 9.2 | 9.2 | 9.1 | 9.1 |
| Phosphorus (mg/dL) | 6.38 | 6.36 | 6.44 | 6.34 | 6.33 | 6.31 |
| Sodium (mmol/L) | 140.8 | 140.8 | 141.0 | 141.4 | 141.1 | 140.8 |
| Potassium (mmol/L) | 5.35 | 5.22 | 5.28 | 5.21 | 5.08 | 5.27 |
| Chloride (mmol/L) | 100.8 | 100.7 | 100.7 | 101.5 | 101.0 | 101.2 |

Mean values per group per timepoint.
