# Supplementary material for: Efficacy and Safety of FX201, a Novel Intra-Articular IL-1Ra Gene Therapy for Osteoarthritis Treatment, in a Rat Model
Source: Hum Gene Ther. 2022 May 16;33(9-10):541–9. doi: 10.1089/hum.2021.131 (PMC9142767; doi:10.1089/hum.2021.131)
Supplement: Supplemental data [file Supp_TableS9.docx]

**Table S9.** **Summary of urinalysis values**

|  | **Males** | | | | | |
| --- | --- | --- | --- | --- | --- | --- |
| Group | 1 | 2 | 3 | 4 | 5 | 6 |
| Dose (GC/dose) | 0 | 0 | 0 | 3.2 x 10^8^ | 3.1 x 10^9^ | 4.3 x 10^10^ |
| Animals per group, *n* | 12 | 12 | 12 | 12 | 12 | 12 |
| **Day 29** |  |  |  |  |  |  |
| Specific gravity | 1.0252 | 1.0228 | 1.0269 | 1.0303 | 1.0273 | 1.0248 |
| pH | 8.13 | 7.42 | 7.83 | 7.29 | 7.50 | 7.75 |
| **Day 92** |  |  |  |  |  |  |
| Specific gravity | 1.0360 | 1.0388 | 1.0262 | 1.0269 | 1.0277 | 1.0338 |
| pH | 7.38 | 7.13 | 7.50 | 7.71 | 7.88 | 7.21 |

Mean values per group per timepoint.
