# Supplementary material for: Efficacy and Safety of FX201, a Novel Intra-Articular IL-1Ra Gene Therapy for Osteoarthritis Treatment, in a Rat Model
Source: Hum Gene Ther. 2022 May 16;33(9-10):541–9. doi: 10.1089/hum.2021.131 (PMC9142767; doi:10.1089/hum.2021.131)
Supplement: Supplemental data [file Supp_TableS10.docx]

**Table S10.** **Summary of microscopic findings on day 29**

|  | **Males** | | | | | |
| --- | --- | --- | --- | --- | --- | --- |
| Group | 1 | 2 | 3 | 4 | 5 | 6 |
| Dose (GC/dose) | 0 | 0 | 0 | 3.2 x 10^8^ | 3.1 x 10^9^ | 4.3 x 10^10^ |
| Animals per group, *n* | 12 | 12 | 12 | 12 | 12 | 12 |
| **Right femorotibial joint examined, *n*** | 12 | 12 | 12 | 12 | 12 | 12 |
| Infiltration, mononuclear cell | (0)^a^ | (2) | (1) | (3) | (2) | (9) |
| Minimal | - | 2 | 1 | 3 | 2 | 7 |
| Mild | - | - | - | - | - | 2 |
| Hypertrophy/hyperplasia; synovium | (0) | (0) | (1) | (2) | (0) | (8) |
| Minimal | - | - | 1 | 2 | - | 7 |
| Mild | - | - | - | - | - | 1 |
| ^a^Numbers in parentheses represent the number of animals with the finding. | | | | | | |

No findings were observed at the day 92 timepoint.

GC, genome copies.
