# Supplementary material for: Efficacy and Safety of FX201, a Novel Intra-Articular IL-1Ra Gene Therapy for Osteoarthritis Treatment, in a Rat Model
Source: Hum Gene Ther. 2022 May 16;33(9-10):541–9. doi: 10.1089/hum.2021.131 (PMC9142767; doi:10.1089/hum.2021.131)
Supplement: Supplemental data [file Supp_Data.docx]

**SUPPLEMENTAL MATERIAL**

**Supplemental Methods**

*HDAd Vectors, Randomization, and Dosing*

FX201 is a nonreplicating, nonintegrating, 29.3 kilobase-HDAd serotype 5 vector that carries a genetic coding sequence for human IL-1Ra. Transcription is controlled by the inflammation-sensitive nuclear factor kappa B (NF-κB)–inducible promoter, comprising five species-conserved NF-κB–binding motif repeats fused to a proximal promoter region of the human endothelial leukocyte adhesion molecule (*ELAM*) gene (**Fig. S2**). HDAd-rat IL-1Ra (HDAd-ratIL-1Ra) carries the rat surrogate of IL-1Ra complementary DNA in a vector backbone identical to FX201.

Organs examined in histopathology assessment that exhibited no HDAd-ratIL-1Ra–related changes following intra-articular (IA) injection of HDAd-ratIL-1Ra included adipose tissue, aorta, bone marrow, brain, epididymis, esophagus, eye, gut-associated lymphoid tissue, adrenal gland, Harderian gland, lacrimal gland, mammary gland, parathyroid gland, pituitary gland, pancreas, skin, small intestine (duodenum, ileum, and jejunum), spinal cord (cervical, lumbar, and thoracic), spleen, stomach, sternum, prostate gland, salivary gland, seminal vesicle gland, thyroid gland, heart, femorotibial joint (left and right), kidney, large intestine (cecum, colon, rectum), liver, lung, lymph node (iliac, mandibular, mesenteric, and popliteal), skeletal muscle, nerve (optic and sciatic), testis, thymus, tongue, trachea, ureter, and urinary bladder.

**Supplemental Results**

Efficacy was also investigated in the Good Laboratory Practice toxicology study where HDAd-ratIL-1Ra was administered 4 weeks following anterior cruciate ligament transection (ACLT) surgery (**Fig. S5**). At day 29 after dose, decreased median composite scores were observed in ACLT-operated rats receiving HDAd-ratIL-1Ra at 3.1×10^9^ genome copies (GC)/dose compared with those receiving vehicle. However, ACLT-operated rats receiving HDAd-ratIL-1Ra at 4.3×10^10^ GC/dose had higher median composite scores compared with those receiving no treatment or vehicle. At day 92 after dose, slightly lower median composite scores were observed only in rats receiving HDAd-ratIL-1Ra at 3.2×10^8^ GC/dose compared with rats receiving no treatment or vehicle.
