# Supplementary material for: Efficacy and Safety of FX201, a Novel Intra-Articular IL-1Ra Gene Therapy for Osteoarthritis Treatment, in a Rat Model
Source: Hum Gene Ther. 2022 May 16;33(9-10):541–9. doi: 10.1089/hum.2021.131 (PMC9142767; doi:10.1089/hum.2021.131)
Supplement: Supplemental data [file Supp_FigS2.docx]

**Figure S2.** FX201 is a helper-dependent adenoviral-based vector containing a human interleukin-1 receptor antagonist (HuIL-1Ra) cassette under the control of an inflammation-responsive nuclear factor-kappa B (NF-κB)–inducible promoter. Furthermore, FX201 is devoid of viral genes and contains human cosmid and human hypoxanthine-guanine phosphoribosyltransferase (HPRT) stuffer sequences. ITR, inverted terminal repeat.
