# Supplementary material for: Efficacy and Safety of FX201, a Novel Intra-Articular IL-1Ra Gene Therapy for Osteoarthritis Treatment, in a Rat Model
Source: Hum Gene Ther. 2022 May 16;33(9-10):541–9. doi: 10.1089/hum.2021.131 (PMC9142767; doi:10.1089/hum.2021.131)
Supplement: Supplemental data [file Supp_FigS5.docx]

**Figure S5.** **HDAd-ratIL-1Ra treatment slightly improved osteoarthritis (OA)-related histopathological features at days 29 and 92 after dose.** Male rats, 8 to 9 weeks old, underwent ACLT surgery and received a single IA injection of HDAd-ratIL-1Ra or vehicle 28 days following surgery. Rats were sacrificed at days 29 and 92 after dose, and joints (*n* = 12 per group at each timepoint) were collected, sectioned, and stained with Safranin-O/Fast Green. Histological changes associated with ACLT-induced OA were assessed via OARSI composite scores at **(A)** day 29 and **(B)** day 92 following IA injection. Mean and median OARSI scores are shown, with whiskers showing the interquartile range (10th and 90th percentiles) and outliers shown as single points (5th and 95th percentiles, where appropriate). Lines within the boxes indicate the median and dotted lines indicate the mean. ACLT-operated rats receiving HDAd-ratIL-1Ra at 3.1×10^9^ GC/dose demonstrated a decrease in composite scores compared with those receiving vehicle at day 29, and rats receiving HDAd-ratIL-1Ra at 3.2×10^8^ GC/dose demonstrated a slight decrease in composite scores compared with those receiving vehicle at day 92. Sham/untreated animals were included as a control. **p* < 0.05; ****p* < 0.001; *****p* < 0.0001, one-way analysis of variance with Tukey’s multiple comparisons test.
