# Supplementary material for: Efficacy and Safety of FX201, a Novel Intra-Articular IL-1Ra Gene Therapy for Osteoarthritis Treatment, in a Rat Model
Source: Hum Gene Ther. 2022 May 16;33(9-10):541–9. doi: 10.1089/hum.2021.131 (PMC9142767; doi:10.1089/hum.2021.131)
Supplement: Supplemental data [file Supp_TableS3.docx]

**Table S3.** **Median OARSI scores for sham- and ACLT-operated rats at week 11**

| **Group** | **Bone and cartilage composite score (interquartile range)** | **Synovial membrane composite score (interquartile range)** |
| --- | --- | --- |
| Sham/untreated | 4.5 (4.0-6.8) | 0.0 (0.0-0.0) |
| ACLT/vehicle | 18.5 (10.8-21.5) | 3.0 (1.5-5.0) |
| ACLT/HDAd-ratIL-1Ra (3.6×10^7^ GC/dose) | 11.5 (9.8-14.3) | 2.0 (0.8-4.0) |
| ACLT/HDAd-ratIL-1Ra (3.1×10^8^ GC/dose) | 9.0 (8.0-10.5) | 1.5 (1.0-2.0) |

ACLT, anterior cruciate ligament transection; GC, genome copies; HDAd, helper-dependent adenovirus; IL-1Ra, interleukin-1 receptor antagonist; OARSI, Osteoarthritis Research Society International. Composite scores for bone and cartilage represent the summed scores for structural changes, Safranin O/fast green staining loss, clone formation, and loss of chondrocytes. For each individual animal, scores for these subcategories are made up of individual scores for four joint compartments: medial femur, lateral femur, medial tibia, and lateral tibia.
